# Neural Correlates of Food Cue Exposure Intervention for Obesity: A Case-Series Approach

**DOI:** 10.3389/fnbeh.2020.00046

**Published:** 2020-04-21

**Authors:** Sieske Franssen, Anita Jansen, Ghislaine Schyns, Karolien van den Akker, Anne Roefs

**Affiliations:** Department of Clinical Psychological Science, Faculty of Psychology and Neuroscience, Maastricht University, Maastricht, Netherlands

**Keywords:** obesity, exposure therapy, functional MRI, case-series, cue reactivity

## Abstract

**Background:**

People with overweight have stronger reactivity (e.g., subjective craving) to food cues than lean people, and this reactivity is positively associated with food intake. Cue reactivity is a learned response that can be reduced with food cue exposure therapy.

**Objectives:**

It was hypothesized that participants after food cue exposure therapy would show reduced neural activity in brain regions related to food cue reactivity and increased neural activity in brain regions related to inhibitory-control as compared to participants receiving a control lifestyle intervention.

**Method:**

Neural activity of 10 women with overweight (BMI ≥ 27 kg/m^2^) in response to individually tailored visually presented palatable high-caloric food stimuli was examined before vs. after a cue exposure intervention (*n* = 5) or a control lifestyle (*n* = 5) intervention. Data were analyzed case-by-case.

**Results:**

Neural responses to food stimuli were reduced in food-cue-reactivity-related brain regions after the lifestyle intervention in most participants, and generally not after the cue exposure therapy. Moreover, cue exposure did not lead to increased activity in inhibitory-control-related brain regions. However, decreased neural activity after cue exposure was found in most participants in the lateral occipital complex (LOC), which suggests a decreased visual salience of high-caloric food stimuli.

**Conclusion:**

Receiving a cue exposure therapy did not lead to expected neural responses. As cue exposure relies on inhibitory learning mechanisms, differences in contexts (e.g., environments and food types) between the intervention setting and the scanning sessions may explain the general lack of effect of cue-exposure on neural activity.

## Introduction

The prevalence of obesity has reached pandemic proportions (World Health Organization, 2018). Overweight people frequently engage in weight loss attempts, but success in the long-term is rare (Bish et al., 2005; Wing and Phelan, 2005). The main cause for obesity is a long-term energy imbalance, in which the number of consumed calories exceeds the number of expended calories for an extended time (Mitchell et al., 2011; Hall et al., 2012). Therefore, developing effective interventions to change behavior and reduce body weight is important. One possible intervention is food cue exposure therapy (CE) (Jansen et al., 2016; van den Akker et al., 2016).

CE aims to reduce food cue reactivity, which is defined as appetitive responding – like increased salivation and self-reported craving – in response to food-associated cues. Food cue reactivity serves as a physiological and psychological preparation for eating (Jansen et al., 2016). Food cues can be internal, such as hunger, satiety, emotions and thoughts, but also external, such as the smell, sight and taste of food, or environmental contexts (Boswell and Kober, 2016; Jansen et al., 2016). As compared to lean people, overweight people have a stronger food cue reactivity (Ferriday and Brunstrom, 2011), which is related to increased food intake (Boswell and Kober, 2016). Food cues become associated with food intake through classical conditioning (Jansen et al., 2016). As soon as food cues are reliable predictors of intake, they will elicit reactivity (Jansen et al., 2011, 2016), which in turn can lead to food intake. In CE, overweight people are repeatedly exposed to food cues while (over)eating is prevented (Bouton, 2004, 2011; Jansen et al., 2011, 2016; van den Akker et al., 2014). Exposure to food cues first *in*creases food cue reactivity, but after prolonged and repeated non-reinforced exposure sessions, this reactivity *de*creases (Jansen et al., 2011, 2016). The CE rationale is that a new association between a food cue and intake is formed: the food cue does *not* predict intake. Importantly, this does not mean that the old association is *un*learned (Bouton, 2004; Jansen et al., 2016). As a result of this inhibitory learning, reactivity to food cues diminishes (extinction). To optimize this inhibitory learning, maximizing “expectancy-violation” is a key element of successful therapy (Craske et al., 2014). Expectancy violation is the reduction in a person’s belief in his/her food-related expectancies (e.g., “If I feel exhausted and chocolate is available, then I will lose control and eat all chocolate”). CE has been shown to be an effective method to reduce food desires and overeating (Boutelle and Bouton, 2015; Jansen et al., 2016; Schyns et al., 2016), and it leads to short-term weight loss (Jansen et al., 2011, 2016; Schyns et al., 2016).

To gain insight in the mechanism of change, examining neural correlates of food cue reactivity may be valuable. A recent review described the following food-cue-reactivity-related brain regions: ventral striatum with nucleus accumbens (NAcc), midbrain, orbitofrontal cortex (OFC), anterior insula (INS), gustatory cortex (GC), lateral occipital cortex (LOC), and somatosensory cortex (SSC) (Giuliani et al., 2018). An increased activity was found in these brain regions when participants viewed high-caloric foods as compared to low-caloric foods or non-food images, and this was also predictive of the amount of food consumed (van der Laan et al., 2011; Smeets et al., 2012; Frankort et al., 2014; Giuliani et al., 2018; Hermann et al., 2019). However, a meta-analysis showed that these effects were quite inconsistent: the concurrence was moderate between studies in the activated clusters to food vs. non-food visual stimuli in healthy-weight participants (van der Laan et al., 2011). As CE intends to reduce food cue reactivity (Jansen et al., 2016), a decreased activity in food-cue-reactivity-related brain regions is expected in the current study.

Additionally, as a candidate-mechanism behind effective CE is inhibitory learning (Boutelle and Bouton, 2015), increased neural activity in inhibitory-control brain regions when processing palatable food stimuli is expected. Inhibitory-control-related brain regions include: dorso- and ventrolateral prefrontal cortex (dlPFC, vlPFC), parietal posterior cortex (PPC), dorsal anterior cingulate cortex (dACC), caudate, pre supplementary motor area (preSMA), and the globus pallidus (GP) (Kober et al., 2010; Giuliani et al., 2018).

In the current study, neural responses in food-cue-reactivity and in inhibitory-control brain regions to individually tailored high-caloric palatable food stimuli were examined pre- and post-CE or a healthy lifestyle (LS) intervention on subject-level (i.e., for each participant separately). During a functional magnetic resonance imaging (fMRI) session, participants were instructed to actively evaluate the taste of the visually presented food stimuli (hedonic focus) or to evaluate the colors of these food stimuli (neutral focus). We hypothesized that CE, as compared to LS, would lead to reduced neural activity in food-cue-reactivity-related brain regions and increased neural activity in inhibitory-control-related brain regions when viewing high-caloric food stimuli, mostly in the hedonic focus condition as this focus is aligned with the experience of craving (Roefs et al., 2018).

## Materials and Methods

### Participants

Ten female overweight participants (BMI: *M* = 32.32 *SD* = 4.43 kg/m^2^, age: *M* = 38.40 *SD* = 10.76 years) from a larger trial (*n* = 45) participated in this study, and were randomly assigned to CE (*n* = 5) or LS (*n* = 5) (van den Akker et al., 2016; Schyns et al., 2019). To overcome the problem of high heterogeneity in neural responses due to individual differences that could occur in small sample sizes (Roiser et al., 2016), data were analyzed on subject-level, as separate cases (for a similar approach see: Hubacher et al., 2015). All participants, except one, were right-handed. Participants were scanned within 2 weeks before and within 2 weeks after intervention.^1^

Inclusion criteria included: female, age between 18 and 60 years, BMI of at least 27 kg/m^2^, no MRI contra-indications and no history of psychiatric or neurological illnesses. The study was approved by the local Ethical Committee. The participants gave written informed consent and were compensated for participation (€ 45).

### Interventions

Interventions were provided by trained students, using a strict protocol, supervised by co-author GS. Both CE and LS consisted of eight individual sessions, scheduled twice per week, during ≈1 month.

During CE, participants performed several food cue exposures with a therapist. The exposure sessions were done in various overeating contexts (e.g., at the laboratory, at home watching television or work). Additionally, participants performed daily exposure exercises on their own at home or at other overeating-associated environmental contexts. LS consisted of four face-to-face sessions alternated with four telephone sessions. LS participants received healthy lifestyle advice, performed mindfulness and power posing exercises, and obtained psychoeducation on body image. For this intervention, daily homework exercises were given on mindfulness and on previous therapy session content. For a detailed description of both interventions see (van den Akker et al., 2016).

### Behavioral Assessments

#### BMI Measurement

Weight and height were measured pre and post-intervention to compute BMI in kg/m^2^.

#### Hunger Assessment

Participants were asked to refrain from eating or drinking (except water) for at least 1 h before the scan-sessions. To check compliance and have an indicative for subjective hunger, self-reported hunger was measured using a 100 mm visual analog scale (VAS), with the question: “How hungry do you feel at this moment?” ranging from 0 (not hungry at all) to 100 (very hungry) at the start of each session. Additionally, participants registered what and at what time they had eaten last.

#### Expectancy Violation

Eight food-cue-associated eating beliefs were rated on perceived expectancy if an associated cue would be followed by eating. Expectancies were measured pre- and post-intervention using 100 mm VAS, with a higher score reflecting a greater perceived expectancy of eating (see methodology paper for details: van den Akker et al., 2016).

### Stimuli

#### Individual Stimulus Selection

Food stimuli used in the fMRI experiment were individually tailored. Each participant selected their five most palatable food items from a list of 33 high-caloric food items in an online questionnaire that was completed ≈1 week before the first scanning session. She then rated the selected stimuli on 10-point scales ranging from 1 (not palatable at all) to 10 (very palatable).

#### Stimulus Presentation

For each of the five chosen palatable food items, two different pictures were included in the fMRI stimulation protocol, to avoid visual adaptation by seeing the same picture too often. Pictures were presented as pop-out high-resolution colored images on a light gray background (RGB: 191 191 191; CKYM: 25 20 20 0) in the center of a black screen covering a visual angle of ≈12°.

### Experimental Task

#### Attentional Focus Manipulation

The participant performed a fast-paced 1-back task in each functional run to induce an attentional focus (hedonic vs. neutral). During the 1-back task, the participant compared each presented food picture (starting from the 2nd presented picture) to the previously presented picture, and indicated whether the presented food was more or less palatable than the previous one (hedonic focus), or whether the picture contained more or fewer colors than the previous one (neutral focus). Each food stimulus was presented for 500 ms, with an inter-stimulus interval (ISI) as a response window of 1,500 ms. The participant’s responses were registered using a buttonbox, with a right index finger press for “fewer” and a right middle finger press for “more.”

#### fMRI Stimulation Protocol

The fMRI task consisted of four runs. In each run, six different conditions were presented, but for the current study only two conditions were relevant and included in the analyses: blocks with palatable high-caloric food stimuli – neutral focus (PAL-NEU) and blocks with palatable high-caloric food stimuli – hedonic focus (PAL-HED). Ten blocks were presented seven times in a randomized order with 12 stimuli each, across the four functional runs. Prior to each block, a cue-word “taste” or “color” was presented for 1 s to inform the participant which attentional focus to apply. Blocks lasted 24 s and were always followed by a 20 s rest block (fixation cross). Total functional scanning time was ≈35 min.

### MRI Data Acquisition

Images were acquired on a 3 Tesla MRI scanner (Magnetom Prisma Fit, Siemens Medical Systems) using a 64-channel head/neck coil. Functional (T_2_^∗^-weighted) images were acquired using multiband gradient echo-planar imaging in an axial interleaved order (Feinberg et al., 2010) with the following settings: TR = 2,000 ms, TE = 30 ms, flip angle = 77°, FOV = 208 × 124 mm^2^, and voxel size of 2 × 2 × 2 mm^3^. To ensure whole brain coverage, slices were acquired in a backward tilted direction of ≈15 degrees to the transversal – coronal line. As anatomical scan, a high-resolution, three-dimensional (3D) T_1_-weighted MPRAGE scan was acquired, with the following settings: TR = 2,250 ms, TE = 2.21 ms, flip angle = 9°, FOV = 256 × 192 mm^2^, and voxel size 1 × 1 × 1 mmł and had a duration of ± 5 min.

### fMRI Data Analysis

#### Preprocessing

Analyses were performed using SPM12 (Statistical Parametric Mapping, London, United Kingdom) and Matlab version 8.3.0.532 (R2014a). Functional images were slice-time corrected, realigned, co-registered, normalized using unified brain segmentation, and spatially smoothed using a Gaussian kernel of 6 mm full width at half-maximum (FWHM). Preprocessed functional volume time series were used for statistical analysis.

#### Statistical Analysis

To compare the session differences on subject-level, a general linear model (GLM) design matrix was created including the two scan-sessions (pre- and post-intervention) as eight consecutive runs. Each experimental task condition was set as a predictor, which resulted in six predictors of interest per run (with two of interest for the current study). Additionally, six motion and eight run mean intensity predictors of no interest were added to the model as confound regressors. Predictor time courses were obtained using condition box-car shaped waves convolved with a two-gamma ideal hemodynamic response function (HRF).

#### Case Series Approach: First-Level Analysis

To investigate the effects of interest, we computed the following contrasts on subject-level for the high-caloric palatable food conditions: (1) main effect of session (*t*-contrasts A: pre-intervention > post-intervention and B: post-intervention > pre-intervention) and (2) session (pre-intervention vs. post-intervention) ^∗^ attentional focus (neutral vs. hedonic) interaction *F*-contrasts. To extract beta values, each condition of interest was also contrasted against baseline.^2^

#### Region of Interest (ROI) Analysis

We defined a-priori ROI masks for food cue reactivity based on the review of Giuliani and colleagues (Giuliani et al., 2018), including: ventral striatum with NAcc, midbrain, OFC, anterior INS, GC, LOC, and SSC and for inhibitory control, including: dlPFC, vlPFC, PPC, dACC, caudate, preSMA and the GP. The ROI masks were manually generated by using the WFU Pickatlas tool (version 3.0.5) in SPM12.

To correct for multiple comparisons, family-wise error (FWE) correction based on Gaussian random field theory was applied to control for false positives at α = 0.05 on subject-level (Eklund et al., 2016). This method was applied for the statistical maps of the main effects of session, and was combined with a clustersize threshold (k) of three contiguous voxels to only include more robust clusters. For the subtler session ^∗^ attentional focus interaction, uncorrected statistical maps with *p* < 0.001 with *k* = 3 voxels were reported. The MarsBar toolbox^3^ was used to extract beta values in SPM12. For localization and clustersize information of activated clusters, XJview^4^ was used. Figures were created using the MNI brain template from MRIcroGL software package^5^.

## Results

### Behavioral Assessments

An overview of behavioral assessments is provided in Table 1. The time between first and second scan session ranged for CE: 34–43 days and for LS: 36–58 days. Three CE and four LS participants lost weight after the intervention. Hunger-ratings were higher post-intervention than pre-intervention in all CE participants, whereas this was only true for two LS-participants (LS1 and LS5). However, 7 of the 10 participants reported relatively low hunger at post-intervention (scores ≤ 44 on a 0–100 VAS). All participants rated their selected foods as highly palatable (average scores ≥ 8.8 on 10-point scale). Expectancy violation changes (post-intervention – pre-intervention) differed between CE and LS participants. All CE participants showed a higher reduction of eating expectancies after intervention as compared to the LS participants.

**TABLE 1:** Participant characteristics and pre-post intervention behavioral measures and post – pre differences.

			Pre-intervention				Post-intervention				Difference post minus pre			
					
	Age	Palatability rating	BMI	Hunger	Not eaten (min)	Expectancies	BMI	Hunger	Not eaten (min)	Expectancies	BMI	Hunger	Not eaten (min)	Expectancies
CE1	18	10	38.69	0	110	65	38.94	11	120	16	0.26	11	10	−49
CE2	45	9.2	31.33	20	90	63	30.15	67	120	13	–1.18	47	30	−50
CE3	28	9	27.75	53	150	86	26.37	78	90	24	–1.38	25	−60	−62
CE4	33	9	37.42	12	120	88	37.99	29	105	33	0.57	17	−15	−55
CE5	42	9.2	29.19	9	90	80	28.76	36	195	16	–0.43	27	105	−64
Average	**33.2**	**9.28**	**32.88**	**18.8**	**112**	**76.4**	**32.44**	**44.2**	**126**	**20.4**	**−0.43**	**25.4**	**14**	**−56**
LS1	45	9.4	27.24	18	105	58	26.70	44	210	76	–0.54	26	105	18
LS2	46	9.6	37.91	9	120	83	37.27	2	150	48	–0.63	−7	30	−35
LS3	52	8.8	34.26	5	180	31	34.18	1	120	1	–0.07	−4	−60	−30
LS4	29	9	30.74	54	150	76	29.98	20	210	54	–0.76	−34	60	−22
LS5	46	9	34.09	36	300	78	34.15	66	510	51	0.06	30	210	−27
Average	**43.6**	**9.16**	32.85	**24.4**	**171**	**65.2**	**32.46**	**26.6**	**240**	**46**	**−0.39**	**2.2**	**69**	**−19.2**

*BMI, body mass index; CE, Cue exposure intervention; LS, Lifestyle intervention. Hunger and expectancies were scored on a 0–100 mm visual analog scale a higher score reflecting more hunger or greater perceived expectancy. Not Eaten, time passed since eaten last meal.*

### Neural Responses

**FIGURE 1:**
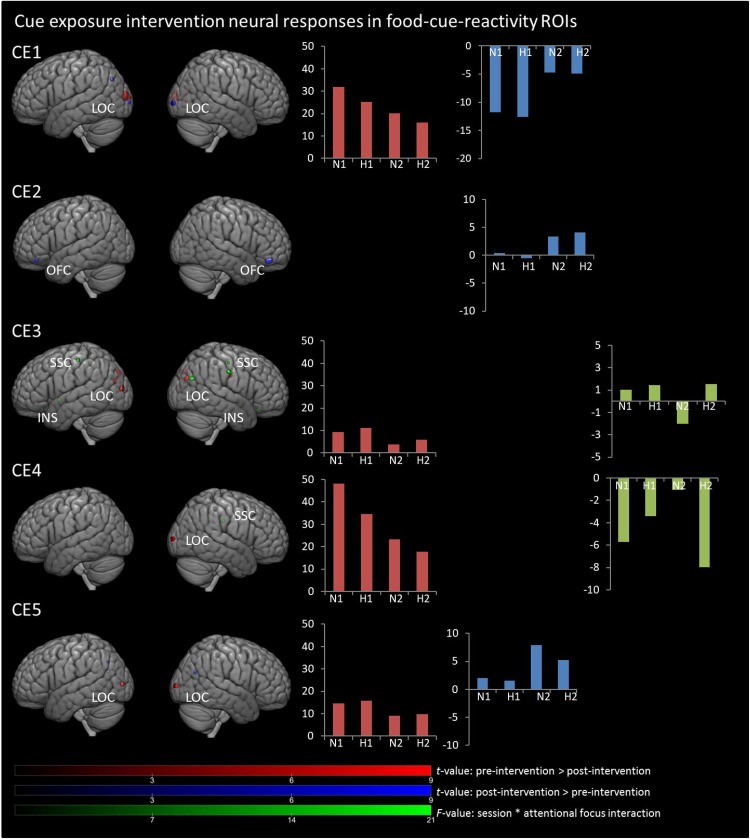
Results from univariate analyses per participant for cue exposure intervention in food-cue-reactivity-ROIs. *t-*maps of significant main effects of session are shown in food-cue-reactivity-ROIs: pre > post-intervention in red, post > pre-intervention in blue (*p* < 0.05 FWE cor) and *F*-map of session * attentional focus interaction (*p* < 0.001 unc.) in green. Bar plots represents mean extracted beta values from the contributing clusters per condition per comparison. N1, neutral attentional focus pre-intervention; H1, hedonic attentional focus pre-intervention; N2, neutral attentional focus post-intervention; H2, hedonic attentional focus post-intervention; LOC, Lateral Occipital Complex; SSC, somatosensory cortex; OFC, orbitofrontal cortex, INS, Insula.

**FIGURE 2:**
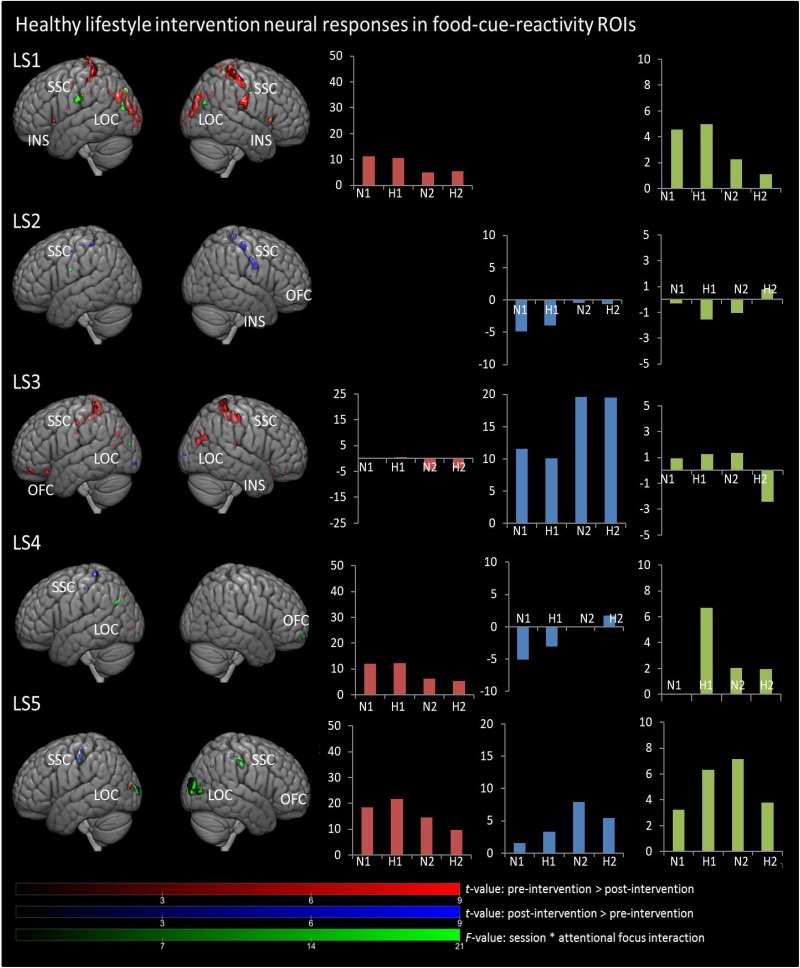
Results from univariate analyses per participant for healthy lifestyle intervention in food-cue-reactivity-ROIs. *t-*maps of significant main effects of session are shown in food-cue-reactivity-ROIs: pre > post-intervention in red, post > pre-intervention in blue (*p* < 0.05 FWE cor) and *F*-map of session * attentional focus interaction (*p* < 0.001 unc.) in green. Bar plots represents mean extracted beta values from the contributing clusters per condition per comparison. N1, neutral attentional focus pre-intervention; H1, hedonic attentional focus pre-intervention; N2, neutral attentional focus post-intervention; H2, hedonic attentional focus post-intervention; LOC, Lateral Occipital Complex; SSC, somatosensory cortex; OFC, orbitofrontal cortex; INS, Insula.

In Figures 1, 2, main effects of session are displayed, as well as the session ^∗^ attentional focus interaction per participant in the food-cue-reactivity-ROIs. Details of each significant cluster can be found in Tables 2, 3.

**TABLE 2:** Significant clusters from univariate analyses per participant for cue exposure intervention in food-cue-reactivity-ROIs.

	Anatomical region	Hemisphere	Clustersize	Peak MNI coordinates			Peak
					
			(No. of voxels)	*x* (mm)	*y* (mm)	*z* (mm)	*F/t*-value
**main effect session: Pre-intervention > Post-intervention**							
CE1	LOC	L	105	−16	−100	10	6.57
	LOC	R	3	28	−92	20	5.34
CE3	LOC	L	23	−40	−90	12	7.06
	LOC	L	6	−38	−80	22	5.08
	LOC	L	3	−26	−84	28	5.31
	LOC	R	22	40	−78	28	5.54
	SSC	R	4	64	−12	32	5.21
	LOC	L	10	−24	−84	38	5.97
	LOC	R	4	34	−78	38	5.17
CE4	LOC	R	14	32	−100	8	6.26
CE5	LOC	R	25	30	−98	6	6.43
	LOC	L	17	−38	−90	6	7.09
**Main effect session: Post-intervention > Pre-intervention**							
CE1	LOC	R	21	30	−100	0	6.13
	LOC	L	6	−26	−100	2	5.15
	LOC	L	10	−38	−76	36	5.29
CE2	OFC	R	48	26	42	−16	7.07
	OFC	L	12	−20	38	−16	5.47
CE5	LOC	R	7	36	−68	24	5.48
	LOC	L	3	−26	−68	40	5.68
Interaction: Session * Attentional focus							
CE3	OFC	R	4	14	28	−18	11.76
	Insula	L	4	−40	4	−4	11.76
	LOC	R	22	46	−70	28	15.48
	SSC	R	13	64	−16	38	12.68
	SSC	R	5	58	−16	52	12.59
	SSC	L	9	−54	−24	54	14.24
CE4	SSC	R	3	66	−24	32	12.62

*L, left; R, right; MNI, Montreal Neurological Institute; LOC, Lateral Occipital Complex; SSC, somatosensory cortex; OFC, orbitofrontal cortex.*

**TABLE 3:** Significant clusters from univariate analyses per participant for healthy lifestyle intervention in food-cue-reactivity-ROIs.

	Anatomical region	Hemisphere	Clustersize	Peak MNI coordinates			Peak
					
			(No. of voxels)	*x* (mm)	*y* (mm)	*z* (mm)	*F/t*-value
**Main effect session: Pre-intervention > Post-intervention**							
LS1	SSC	R	288	50	−28	58	8.19
	LOC	L	271	−26	−86	20	11.32
	LOC	R	188	28	−88	20	9.04
	SSC	L	156	−40	−40	68	9.75
	SSC	R	79	68	−18	24	8.11
	LOC	L	55	−20	−100	2	9.03
	LOC	L	34	−18	−96	14	11.49
	Insula	R	18	40	18	4	6.08
	SSC	R	17	12	−56	72	6.57
	LOC	R	14	38	−74	34	5.94
	SSC	L	14	−22	−30	82	7.00
	Insula	L	9	−40	18	−2	6.08
	LOC	L	8	−32	−95	−8	5.86
	SSC	L	8	−56	−18	26	5.15
	SSC	L	8	−4	−8	48	5.96
	SSC	L	8	−54	−32	52	6.61
	SSC	L	6	−58	−24	32	5.93
	LOC	L	4	−16	−104	−8	9.40
	LOC	L	3	−10	−96	0	5.72
	SSC	L	3	−54	−2	16	5.07
	SSC	L	3	−44	−32	36	5.13
LS3	SCC	R	200	32	−46	70	9.22
	LOC	R	109	44	−72	24	6.43
	SSC	L	105	−28	−48	68	8.51
	SSC	R	78	34	−28	54	7.72
	OFC	L	24	−34	48	16	6.44
	SSC	R	24	54	−24	54	6.11
	SSC	L	20	−56	−18	44	5.64
	OFC	L	19	−32	22	−20	6.56
	SSC	L	19	−18	−46	58	5.98
	SSC	R	14	6	−42	62	7.25
	SSC	R	14	8	−42	74	7.72
	SSC	R	11	6	−36	50	6.19
	LOC	L	10	−40	−72	32	5.40
	Insula	R	8	34	−26	20	5.43
	SSC	L	7	−40	−16	34	5.64
	SSC	L	6	−4	−48	62	5.68
	SSC	L	4	−14	−40	54	5.55
	SSC	L	4	−14	−44	74	6.80
	OFC	L	3	−34	24	−12	5.29
	LOC	L	3	−42	−68	16	5.67
LS4	LOC	L	3	−32	−88	−4	5.06
LS5	LOC	L	43	−28	−90	14	6.90
**Main effect session: Post-intervention > Pre-intervention**							
LS2	SSC	R	64	56	−2	−28	5.79
	SSC	R	37	42	−22	58	6.81
	SSC	L	11	−32	−38	58	5.29
	SSC	R	11	36	−32	68	5.67
	SSC	L	5	28	−26	−56	5.27
	SSC	R	4	28	−26	56	5.19
	Insula	R	3	40	−12	16	5.12
	SSC	L	3	−4	−44	72	5.26
LS3	LOC	L	6	−28	−98	−8	6.42
	LOC	R	4	24	98	4	5.07
LS4	SSC	L	33	−34	−36	68	5.51
	SSC	L	4	−52	−24	50	5.31
LS5	SSC	L	130	−50	−22	44	6.55
Interaction: Session * Attentional focus							
LS1	SSC	L	63	−66	−16	26	29.22
	LOC	L	26	−46	−78	22	15.85
	LOC	R	20	50	−72	26	19.03
	LOC	L	18	−26	−84	40	14.10
	SSC	L	8	−4	−46	70	23.43
	SSC	R	6	60	−10	44	15.61
	Insula	R	4	34	22	0	13.08
	SSC	L	3	−18	−26	80	15.45
LS2	SSC	L	9	−62	−6	24	12.60
	OFC	R	4	6	52	−26	12.74
	OFC	R	3	14	18	−22	13.18
LS3	LOC	L	7	−18	−90	18	12.87
LS4	OFC	R	18	18	64	−12	13.21
	LOC	L	17	−34	−70	34	12.15
LS5	LOC	R	224	36	−90	12	21.36
	SSC	R	68	48	−22	38	22.93
	LOC	L	47	−26	−100	10	15.41
	LOC	L	35	52	−78	6	16.79
	SSC	L	18	−38	−24	56	13.19
	SSC	L	17	−52	−20	44	13.48
	LOC	R	8	38	−76	14	11.73
	LOC	R	4	32	−62	38	12.11
	SSC	R	4	50	−32	52	12.65
	OFC	R	3	30	26	−18	11.06

*L, left; R, right; MNI, Montreal Neurological Institute; LOC, Lateral Occipital Complex; SSC, somatosensory cortex; OFC, orbitofrontal cortex.*

#### Main Effects Session

Contrary to our hypothesis, food-cue-reactivity-related activity was not substantially reduced after intervention for CE participants. In fact, LS participants showed reductions in more ROIs (e.g., SSC, INS, LOC, and OFC; see participants LS1 and LS3), and involved clusters were larger. However, reduction in beta values was larger in those clusters that changed significantly from pre- to post-intervention in CE participants. Note that these clusters were substantially smaller and localized in the LOC solely (see participants: CE1, CE3, CE4, and CE5). To examine opposite effects, we also compared post-intervention > pre-intervention contrasts. Here, unexpectedly, the CE participants showed also *in*creased activation in small clusters in the LOC (see: CE1 and CE5) and in the OFC (CE2) after intervention. LS participants showed an increase in activity in the SSC (LS2, LS4 and LS5) and in the LOC (LS3).

#### Session ^∗^ Attentional Focus Interaction

As with the main effect of session, the interaction effect was also mainly observed in LS participants. Analyses of the interaction yielded significant clusters for all LS participants. Here, four LS participants (LS1, LS3, LS4, and LS5) showed a larger reduction from pre to post with the hedonic focus than with the neutral focus in the right INS and OFC and bilaterally in the SSC and LOC. For participant LS2 this was reversed, activity in food-cue-reactivity-ROIs (right OFC and left SSC) was increased from pre to post with the hedonic focus. Two CE participants showed an interaction effect, where participant CE4 showed a more reduced activation pre to post in the hedonic focus than in the neutral focus in a very small cluster in the right SCC. Participant CE3 showed more robust clusters, involving the INS and the SCC with an unexpectedly larger reduction in activation from pre to post in the neutral focus condition than the hedonic focus.

We also compared neural activity per participant in inhibitory-control-ROIs. Tests of the main- and interaction-effects did not lead to any meaningful results for the CE intervention either. The clusters of neural activity per participant can be found in Supplementary Tables S1, S2.

## Discussion

Contrary to our hypothesis, the results showed that for these cases a cue exposure intervention did not lead to a significantly stronger reduction in neural activity in brain regions related to food-cue-reactivity, in response to visual high-caloric palatable food stimuli, as compared to the participants that received a lifestyle intervention. In fact, most participants’ reductions in neural activity in food-cue-reactivity-related brain regions were more pronounced and more widespread after a lifestyle intervention and mostly with a hedonic attentional focus. When comparing activity in inhibitory-control-related brain regions on subject-level, no meaningful results were observed.

Surprisingly, the expected reduced activity was more apparent in LS participants (in e.g., SSC, INS, OFC, and LOC). During the intervention, LS participants received education on dieting and healthy weight loss and on nutrients and energy balance (van den Akker et al., 2016). This could have raised awareness of negative health aspects of high caloric foods, which may have contributed to participants’ reduced neural responses to high-caloric foods. This interpretation aligns well with previous studies, showing that focusing on negative health aspects can control reward-related activity to visually presented high-caloric food stimuli (Hollmann et al., 2012; Siep et al., 2012). Although participants were instructed during scanning to attend to the hedonic aspects of the foods presented, this lifestyle training may have interfered with this hedonic focus during the post-intervention scanning-session by increasing awareness of negative health aspects.

Unexpectedly, CE did not lead to a significant reduction of neural activity in most cases in food-cue-reactivity-related brain regions. Behavioral outcomes showed that self-reported expectancy violations did improve specifically for the CE participants. Also, hunger was higher for all CE participants at post-intervention measurement. However, these CE-related behavioral effects could not be meaningfully related to post-pre intervention patterns of neural activity. These neural pre-post intervention findings could be the consequence of participants learning a new inhibitory association (the cue does *not* predict intake) during food cue exposure, which then exists next to the original disinhibiting association (the cue does predicts intake) (Jansen et al., 2016). That is, the food-cue-intake association is not erased, and therefore food cues might still trigger neural activity in food-cue-reactivity-ROIs. However, also in inhibitory-control-related brain regions, no strong increased neural activity in CE participants was found after intervention in these inhibitory-control regions.

Important to realize is that inhibitory learning during extinction is context-dependent and food-specific (Bouton, 2004, 2011; Jansen et al., 2016). Both the context (fMRI scanner vs. a laboratory room, participants’ home and other relevant contexts) and the food stimuli differed between the current fMRI measurement and the intervention setting. Furthermore, a CE intervention only led to reduced consumption of the exposed foods, but not of other foods (Schyns et al., 2016, 2018, 2019). So, there was no generalization to other foods. In an earlier study (Frankort et al., 2014), we did observe a reduction in neural activity in food-cue-reactivity-related brain regions after cue exposure. Importantly, here, the cue exposure and measurement of neural activity both took place in the scanner while using the same food stimuli throughout (i.e., chocolate) (Frankort et al., 2014). Taken together, these findings underline the importance of considering context and the food-specificity of cue exposure while examining neural responses.

In line with our hypothesis, the current study showed reduced activation in the lateral occipital complex (LOC) in four participants after CE. The LOC was identified, in a meta-analysis comparing visual food to non-food stimuli, as one of the main brain regions involved in visual food cue processing (van der Laan et al., 2011). The decreased LOC activity may reflect a decrease in visual saliency of the palatable high-caloric foods as a result of CE. As this decreased LOC activation was specifically found for the CE participants, it therefore might be a precursor for extinction.

A limitation of this study is that due to the inclusion of only female participants and the case-series analyses approach, it is hard to translate the current results to a group-intervention effect, or to a broader population (i.e., males). Results should be interpreted with caution. Data was analyzed with a-priori defined ROI masks, which reduces the between-subjects variability of activation locations (Ziauddeen et al., 2012) and makes interpreting and comparing findings more reliable. The scanning-protocol pre- and post-intervention was kept exactly the same, which made it a strong within-subject design, and therefore the current study might give interesting leads for conducting a group-level future study. Future research needs to replicate these findings, and investigate whether neural changes induced by a lifestyle intervention are related to concurrent and future weight change.

## Data Availability Statement

All datasets generated for this study are included in the article/Supplementary Material.

## Ethics Statement

The studies involving human participants were reviewed and approved by the local Ethical Committee of the faculty of Psychology and Neuroscience, Maastricht University. The patients/participants provided their written informed consent to participate in this study.

## Author Contributions

SF, AR, and AJ designed the study. SF collected the data and analyzed the data. GS and KA designed and coordinated the interventions. SF and AR wrote the manuscript. AJ, GS, and KA gave feedback on the manuscript. All authors approved the final version.

## Conflict of Interest

The authors declare that the research was conducted in the absence of any commercial or financial relationships that could be construed as a potential conflict of interest.

## Footnotes

1 Note that these pre-intervention fMRI data was also included in a previous study (Franssen et al., 2020).

2 For participant 2 of the LS group only three runs were acquired in the pre-intervention session due to set-up problems. Therefore, also only three runs of the post-intervention session were included for this participant. This was done to be able to balance the conditions in the GLM for defining contrasts. For the post-intervention session, we selected the three runs with best data quality (i.e., least movement of participant during scanning).

3 http://marsbar.sourceforge.net/

4 http://www.alivelearn.net/xjview/

5 http://www.mccauslandcenter.sc.edu/mricro/mricrogl/
