# Supplementary material for: Neural Correlates of Food Cue Exposure Intervention for Obesity: A Case-Series Approach
Source: Front Behav Neurosci. 2020 Apr 21;14:46. doi: 10.3389/fnbeh.2020.00046 (PMC7187770; doi:10.3389/fnbeh.2020.00046)
Supplement: Supplementary file 1 [file Table_1.docx]

**Supplementary Material**

**S1. Significant clusters from univariate analyses per participant for cue exposure intervention in inhibitory-control-ROIs.**

|  | **Anatomical region** | **Hemisphere** | **Clustersize** | **peak MNI coordinates** | | | **peak** |
| --- | --- | --- | --- | --- | --- | --- | --- |
|  |  |  | (num. | x(mm) | y(mm) | z(mm) | *F/t*-value |
|  |  |  | of voxels) |  |  |  |  |
| **Main effect session: pre intervention > post intervention** | | | | | | | |
| **CE3** | PPC | R | 201 | 13 | -70 | 64 | 6.59 |
|  | vlPFC | R | 40 | 40 | 64 | 0 | 6.64 |
|  | PPC | L | 37 | -10 | -76 | 52 | 5.66 |
|  | preSMA | R | 30 | 14 | -6 | 76 | 5.95 |
|  | vlPFC | L | 16 | -28 | 60 | 28 | 5.82 |
|  | preSMA | R | 9 | 4 | 6 | 46 | 5.11 |
|  | preSMA | L | 8 | -32 | -8 | 66 | 6.08 |
|  | PPC | L | 5 | -30 | -60 | 60 | 5.07 |
|  | PPC | R | 4 | 42 | -42 | 56 | 5.49 |
|  | PPC | L | 3 | -22 | -52 | 70 | 5.37 |
| **CE5** | PPC | L | 21 | -22 | -66 | 70 | 6.47 |
| **Main effect session: post intervention > pre intervention** | | | | | | | |
| **CE2** | preSMA | R | 149 | 12 | 12 | 72 | 6.95 |
|  | preSMA | R | 42 | 32 | 8 | 66 | 7.93 |
|  | preSMA | L | 21 | -2 | 10 | 60 | 5.54 |
|  | preSMA | R | 14 | 6 | 18 | 48 | 5.89 |
|  | preSMA | L | 10 | -28 | 4 | 70 | 5.83 |
|  | preSMA | L | 3 | -14 | 2 | 76 | 5.69 |
| **Interaction: session * attentional focus** | | | | | | | |
| **CE1** | vlPFC | R | 3 | 38 | 64 | 2 | 13.07 |
| **CE2** | preSMA | R | 8 | 32 | 2 | 62 | 11.63 |
|  | dlPFC | R | 5 | 50 | 34 | 16 | 11.78 |
| **CE3** | dACC | R | 20 | 12 | 34 | 24 | 14.32 |
| **CE4** | dlPFC | R | 12 | 6 | 50 | 48 | 14.81 |
|  | vlPFC | L | 7 | -12 | 64 | 12 | 15.94 |
|  | vlPFC | R | 4 | 30 | 62 | 18 | 12.49 |
|  | vlPFC | R | 3 | 42 | 50 | 4 | 11.89 |
|  | dlPFC | R | 3 | 24 | 48 | 42 | 11.09 |

Abbreviations: L = left, R = right, MNI = Montreal Neurological Institute, dlPFC = dorsolateral prefrontal cortex, vlFPC = ventrolateral prefrontal cortex, PPC = parietal posterior cortex, dACC = dorsal anterior cingulate cortex and preSMA = pre supplementary motor area

**S2. Significant clusters from univariate analyses per participant for healthy lifestyle intervention in inhibitory-control-ROIs.**

|  | **Anatomical region** | **Hemisphere** | **Clustersize** | **peak MNI coordinates** | | | **peak** |
| --- | --- | --- | --- | --- | --- | --- | --- |
|  |  |  | (num. | x(mm) | y(mm) | z(mm) | *F/t*-value |
|  |  |  | of voxels) |  |  |  |  |
| **Main effect session: pre intervention > post intervention** | | | | | | | |
| **LS1** | PPC | L | 192 | -20 | -62 | 72 | 10.72 |
|  | PPC | R | 104 | 14 | -54 | 68 | 7.43 |
|  | preSMA | R | 77 | 26 | 6 | 64 | 6.70 |
|  | preSMA | L | 70 | -2 | 2 | 74 | 6.55 |
|  | preSMA | L | 53 | -20 | 2 | 72 | 6.53 |
|  | dlPFC | R | 52 | 48 | 46 | 12 | 8.02 |
|  | PPC | L | 32 | -12 | -82 | 47 | 7.95 |
|  | preSMA | L/R | 26 | 2 | 10 | 44 | 6.40 |
|  | PPC | L | 19 | -20 | -66 | 56 | 6.02 |
|  | vlPFC | R | 18 | 40 | 18 | 4 | 6.08 |
|  | preSMA | R | 18 | 30 | -8 | 70 | 6.59 |
|  | vlPFC | L | 10 | -54 | 14 | 26 | 5.64 |
|  | preSMA | L | 7 | -26 | -6 | 54 | 6.05 |
|  | dlPFC | L | 6 | -46 | 30 | 14 | 5.24 |
|  | PPC | R | 6 | 8 | -80 | 50 | 5.55 |
|  | Caudate | R | 5 | 14 | -8 | 18 | 5.79 |
|  | vlPFC | R | 5 | 56 | 14 | 26 | 6.54 |
|  | vlPFC | L | 3 | -40 | 16 | 0 | 5.64 |
|  | dlPFC | R | 3 | 42 | 20 | 24 | 4.93 |
| **LS3** | PPC | R | 389 | 14 | -66 | 64 | 9.00 |
|  | PPC | L | 177 | -16 | -48 | 74 | 9.23 |
|  | vlPFC | L | 55 | -48 | 24 | -8 | 6.50 |
|  | dlPFC | L | 30 | -20 | 34 | 42 | 6.19 |
|  | preSMA | R | 30 | 20 | -10 | 72 | 6.51 |
|  | vlPFC | L | 29 | 0 | 50 | 16 | 5.96 |
|  | PPC | L | 28 | -22 | -68 | 58 | 6.40 |
|  | dlPFC | L | 27 | -8 | 56 | 34 | 6.62 |
|  | preSMA | R | 27 | 22 | 4 | 68 | 6.71 |
|  | dlPFC | R | 21 | 20 | 64 | 8 | 6.04 |
|  | preSMA | R | 21 | 4 | -4 | 68 | 5.69 |
|  | preSMA | L | 19 | -12 | 18 | 64 | 5.82 |
|  | vlPFC | R | 15 | 60 | 28 | 4 | 6.71 |
|  | dlPFC | L | 12 | -22 | 54 | 12 | 5.81 |
|  | dlPFC | R | 11 | 54 | 42 | 6 | 6.37 |
|  | preSMA | L | 10 | -24 | 10 | 64 | 5.59 |
|  | dlPFC | L | 7 | -10 | 42 | 22 | 5.46 |
|  | PPC | L | 7 | -16 | -78 | 54 | 5.58 |
|  | PPC | L | 6 | -18 | -50 | 60 | 5.54 |
|  | preSMA | R | 6 | 32 | -12 | 66 | 5.44 |
|  | PPC | R | 5 | 42 | -46 | 64 | 5.76 |
|  | vlPFC | R | 4 | 58 | 22 | -6 | 5.85 |
| **LS4** | preSMA | R | 53 | 34 | 0 | 68 | 6.59 |
| **Main effect session: post intervention > pre intervention** | | | | | | | |
| **LS1** | vlPFC | R | 23 | 34 | 64 | 0 | 6.84 |
|  | preSMA | R | 19 | 4 | 40 | 42 | 5.58 |
|  | vlPFC | R | 3 | 4 | 60 | 0 | 5.18 |
| **LS2** | dACC | R | 95 | 6 | -8 | 50 | 6.21 |
|  | preSMA | R | 13 | 6 | -10 | 78 | 5.54 |
|  | dlPFC | L | 3 | -20 | 30 | 54 | 5.22 |
| **LS3** | PPC | L | 70 | -26 | -66 | 54 | 7.10 |
|  | vlPFC | R | 48 | 48 | 42 | -2 | 6.55 |
|  | vlPFC | R | 25 | 54 | 18 | 6 | 6.10 |
| **LS5** | vlPFC | R | 7 | 8 | 68 | 14 | 5.89 |
| **Interaction: session * attentional focus** | | | | | | | |
| **LS1** | PPC | L | 48 | -14 | -80 | 46 | 18.87 |
|  | vlPFC | R | 27 | 56 | 26 | 10 | 17.06 |
|  | dlPFC | R | 22 | 26 | 36 | 60 | 14.26 |
|  | vlPFC | R | 15 | 52 | 47 | 10 | 19.91 |
|  | dlPFC | R | 15 | 12 | 66 | 28 | 21.83 |
|  | preSMA | R | 15 | 12 | 16 | 70 | 25.30 |
|  | vlPFC | R | 13 | 14 | 70 | 10 | 13.37 |
|  | vlPFC | L | 13 | -58 | 22 | 24 | 17.05 |
|  | PPC | L | 8 | -34 | -72 | 54 | 12.23 |
|  | vlPFC | L | 7 | -64 | 16 | 10 | 19.93 |
|  | PPC | R | 6 | 26 | -80 | 50 | 13.90 |
|  | vlPFC | R | 5 | 34 | 62 | 14 | 14.77 |
|  | vlPFC | L | 4 | -18 | 66 | 20 | 12.24 |
|  | dlPFC | L | 4 | -20 | 42 | 48 | 12.36 |
|  | preSMA | L | 4 | -24 | 6 | 68 | 13.02 |
|  | preSMA | R | 4 | 16 | -14 | 76 | 13.12 |
|  | vlPFC | L | 3 | -48 | 34 | -2 | 11.73 |
| **LS3** | dlPFC | L | 24 | -50 | 50 | 2 | 17.00 |
|  | dlPFC | R | 16 | 12 | 62 | 26 | 17.92 |
|  | PPC | L | 15 | -28 | -76 | 50 | 15.71 |
|  | vlPFC | R | 13 | 22 | 64 | 0 | 16.27 |
|  | vlPFC | R | 13 | 18 | 68 | 8 | 19.35 |
|  | vlPFC | L | 9 | -14 | 68 | 4 | 14.48 |
|  | vlPFC | L | 5 | -22 | 64 | 18 | 11.63 |
|  | dlPFC | L | 4 | -20 | 52 | 38 | 14.83 |
| **LS4** | PPC | L | 79 | -30 | -60 | 66 | 20.71 |
|  | vlPFC | R | 71 | 42 | 38 | -2 | 18.91 |
|  | vlPFC | R | 42 | 56 | 22 | 16 | 14.99 |
|  | vlPFC | R | 33 | 20 | 70 | 8 | 15.79 |
|  | dlPFC | L | 28 | -46 | 48 | 0 | 13.07 |
|  | preSMA | L | 24 | -2 | 20 | 44 | 14.37 |
|  | vlPFC | R | 20 | 46 | 20 | 8 | 15.85 |
|  | vlPFC | L | 12 | -58 | 18 | 20 | 13.32 |
|  | dlPFC | R | 10 | 40 | 44 | 2 | 11.94 |
|  | vlPFC | L | 8 | 0 | 66 | 12 | 12.57 |
|  | PPC | R | 8 | 40 | -52 | 64 | 14.10 |
|  | preSMA | R | 4 | 14 | 26 | 60 | 12.23 |
| **LS5** | PPC | L | 70 | -26 | -66 | 54 | 7.10 |
|  | vlPFC | R | 7 | 8 | 68 | 14 | 5.89 |

Abbreviations: L = left, R = right, MNI = Montreal Neurological Institute, dlPFC = dorsolateral prefrontal cortex, vlFPC = ventrolateral prefrontal cortex, PPC = parietal posterior cortex, dACC = dorsal anterior cingulate cortex and preSMA = pre supplementary motor area
